# IoT Based Heart Activity Monitoring Using Inductive Sensors

**DOI:** 10.3390/s19153284

**Published:** 2019-07-26

**Authors:** Adrian Brezulianu, Oana Geman, Marius Dan Zbancioc, Marius Hagan, Cristian Aghion, D. Jude Hemanth, Le Hoang Son

**Affiliations:** 1 Faculty of Electronics, Telecommunications and Information Technology, “Gheorghe Asachi” Technical University from Iaşi, Iaşi 700050, Romania; 2 Department of Health and Human Development, Stefan cel Mare University, Suceava 720229, Romania; 3 Department of ECE, Karunya Institute of Technology and Sciences, Coimbatore, Tamilnadu 641114, India; 4 Division of Data Science, Ton Duc Thang University, Ho Chi Minh City 700000, Vietnam; 5 Faculty of Information Technology, Ton Duc Thang University, Ho Chi Minh City 700000, Vietnam

**Keywords:** IoT, UH, inductive sensor, textile-based sensor, heart rate sensing, respiration sensing, inductive to number convertor

## Abstract

This paper presents a system dedicated to monitoring the heart activity parameters using Electrocardiography (ECG) mobile devices and a Wearable Heart Monitoring Inductive Sensor (WHMIS) that represents a new method and device, developed by us as an experimental model, used to assess the mechanical activity of the hearth using inductive sensors that are inserted in the fabric of the clothes. Only one inductive sensor is incorporated in the clothes in front of the apex area and it is able to assess the cardiorespiratory activity while in the prior of the art are presented methods that predict sensors arrays which are distributed in more places of the body. The parameters that are assessed are heart data-rate and respiration. The results are considered preliminary in order to prove the feasibility of this method. The main goal of the study is to extract the respiration and the data-rate parameters from the same output signal generated by the inductance-to-number convertor using a proper algorithm. The conceived device is meant to be part of the “wear and forget” equipment dedicated to monitoring the vital signs continuously.

## 1. Introduction

Nowadays, the Internet of Things (IoT) through which a network of smart objects work together in collecting and analyzing big collections of data and autonomously perform actions is becoming real, thanks to the machine-to-machine (M2M) technology [1]. Globally, M2M connections will grow nearly threefold, from 4.9 billion in 2015 to 12.2 billion by 2020, accounting for nearly half (46%) of all connected devices, including medical devices. “For 2020, the installed base of Internet of Things devices is forecast to grow to almost 31 billion worldwide [1]”. The medical area will grow fivefold, the fastest, from 144 million connections in 2015 to 729 million in 2020 [1]. There are many applications of IoT in medicine, including in electrocardiography [2,3,4,5,6,7,8,9,10,11,12,13,14,15,16,17].

Chronic disease management is important for the self-management of health and the IoT concept plays a significant role. Personal medical devices need two features: Applications that use network protocols and intelligent services to achieve them. However, most have only one function: To indicate data and save them temporarily. This paper suggests a smart health service model that gives a concrete response to an individual. To do this, we have introduced a collaborative protocol that transfers risk factors between IoT and personal medical devices. “Ubiquitous Health” (UH) is a model where individual medical data were measured by ubiquitous (UHD) personal medical devices, then sent to a dedicated server to provide answers to medical staff and patients. The intelligent clothes represent a paradigm that gains a large area in people’s life. The technological progress determines an easy implementation of models in wearable devices, the “wearability” being a needed feature of intelligent clothes [17,18,19,20,21,22,23,24,25,26,27,28].

“Smart textiles are defined as textile products, such as fibers, filaments, and yarns together with woven, knitted or non-woven structures, which can interact with the environment/user” [21]. This interaction implies changes in certain values of electric or electromagnetic fields modifying the values of physical parameters such as resistances, capacitances, and inductances.

We can talk about capacitive smart textiles by applying conductive paint on a fabric in a configuration of capacitor electrodes [22,23,24], the capacitances values being determined by a relative position of an object related to the surface of the electrodes. The smart textile concepts imply two directions of development: Implementing of passive components like resistors (by conductive tissues), inductances (by coil shape), and capacitive, but also, we should take into account the possibility to implement active components like transistors. In this case, we can imagine a configuration of the circuits distributed on the surface and in the volume of the clothes.

A capacitive sensorial structure was conceived in order to detect the roll-pitch-yaw rotations of the neck and hands. The capacitive sensor was positioned on the collar and on the sleeves of the clothes and an accelerometric sensor was dedicated to determining the neck and trunk movements.

The accelerometric sensor is placed on the collar, in the cervical zone, close to the capacitive sensors [24]. An inductive proximity sensor is dedicated to monitoring the mechanical heart activity by inserting a planar inductive sensor in the fabric of the clothes in front of the apex zone. The displacements of the chest tissues during systole and diastole phases are seen as inductive changing values that are converted into digital numbers.

The paper presents an ECG (Electrocardiography)-based monitoring system [23] using an acquisition device of the ECG signals to which electrodes are attached using wires that will be in contact with the patient’s skin (over the sampling period) and also an accelerometer device.

**Figure 1:**
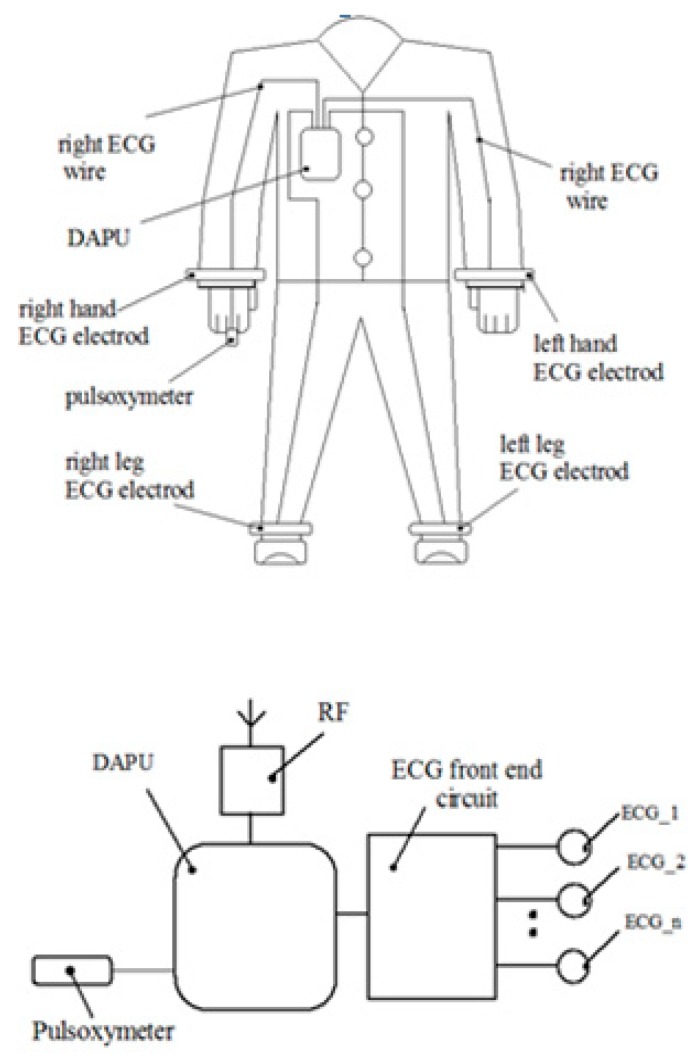
Smart clothing for Electrocardiography ECG [23].

This system supports the following functions: ECG signal acquisition, heart rate monitor, respiration rate measurement based on impedance pneumography (see Figure 1). The device dedicated to monitoring the heart activity consists of a planar inductive sensor that is inserted in the fabric and is connected to a LDC circuit (Inductive to digital convertor), of a microcontroller that gathers the inductive data and sends them by an RF module to a server. One inductive sensor only is incorporated in the clothes in front of the apex area and it is able to assess the cardiorespiratory activity while in the prior of the art are presented methods that predict sensors arrays which are distributed in more places of the body.

The heart activity monitoring using wearable biosensors has as a goal the continuous assessment of cardiovascular parameters [23,24,25]. An advanced photoplethysmographic technique is presented in [25].

This technique adopts as a monitoring device a ring that incorporates a sensorial structure which is able to measure the heart parameters such as heart rate, oxygen saturation, and heart rate variability. “Wearable electrocardiogram systems represent the most mature WBS technology.” [27].

The same devices are used in order to store ECG signals for a determined time period being able to generate outputs for heart rate and respiration. Dedicated integrated circuits with these functionalities have been developed (we would like to mention here ADAS1000 from Analog Devices).

The movements of the tissues are detected by acceleration sensors which could be used in order to identify and to reduce the motion artifacts [28].

A planar inductive sensing element (“flat spiral”) which is inserted in the clothes in order to detect the vital parameters (heart-rate and respiration) is presented in [29]. The simulation of the theoretical model was performed by COMSOL Multiphysics (COMSOLAB, U.S.A.), magnetic field model. The principle of operation consists of two stages: “The first step was to evaluate the induced eddy current by the excitation of an external coil, and the second step was to evaluate the induced magnetic fields by an eddy current.” [29]. The presence of a coil on a fabric offers the privilege to use the coil as an antenna with two functionalities: Sensing element and data transfer element [28]. Another functionality could be charging coil for the accumulators when the clothes are not worn.

In [30] a method dedicated to monitoring cardiorespiratory activity is presented that is based on the magnetic induction technique. The paper mentions that the capacitance between the coil and the body (denominated C_stray_) did not influence the output signal, but during the physical activity this distance is not constant, therefore, in our opinion, the distance between the sensor and the thoracic plan has a large influence on the output signal value. The differences between [30] and our paper consist of:

(1) In [30] more sensors are used in order to determine the changes of the thoracic impedances, these changes being caused by cardiorespiratory activity. In this paper, we used only a sensor that is positioned in front of the apex and assesed the cardiorespiratory activity based on the changing of the thoracic impedance and of the distance between the sensor and thoracic surface. These parameters influence the inductance value of the sensor, this value is measured using a very sensitive inductance to digital convertor (LDC1612).
(2) In [30] inductive sensors based on the microcontroller MSP430F5435A are conceived and implemented. In this work, we used a dedicated inductance-to-digital convertor (LDC1612) that has very good sensitivity and measurement resolution (up to 28 bits).

In [31] a device for monitoring the pulse by measurement the bioimpedance of the thumb based on magnetic induction principle is described. An inductive sensor is placed on the thumb, as a ring, this method is an alternative for the photoplethysmographic sensors. This monitoring technique is able to determine the pulse only.

## 2. IoT and GreenCardio Platform

GreenCardio© [14] is a dedicated system to monitor the heart activity parameters using various mobile or wearable devices that are connected to a cloud. The system consists of a set of heart monitoring devices (wearable heart monitoring inductive sensor WHMIS, mobile electrocardiograph device, MED, being available to connect other kinds of peripherals), a data server and an application that represents a software platform for remote ECG investigation that is able to collect, centralize and diagnose ECG investigations.

**Figure 2:**
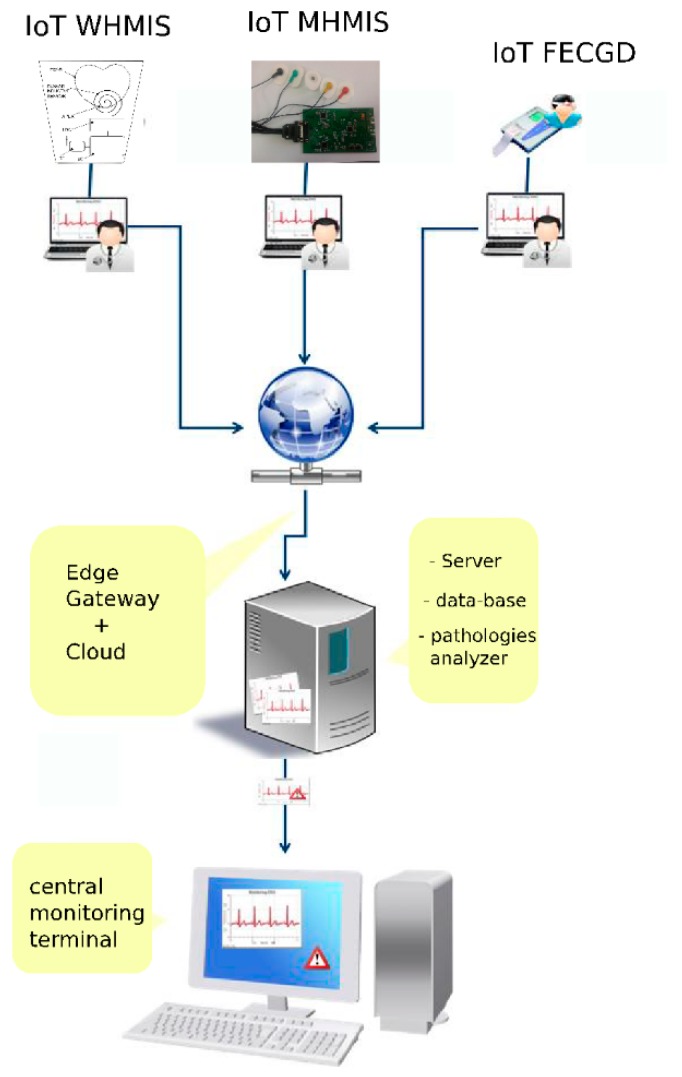
GreenCardioIoT system [14].

The application runs as follows [14]:

(i) In the family doctor’s office, as a separate module (FD, desktop), installed on a desktop computer or laptop that is connected to an ECG device, information regarding the ECG investigations will be stored initially on the local hard drive in a data base. Based on internet connection availability, the results of the ECG investigations will be transmitted to a central server to be parallel processed and monitored;
(ii) On a central server (collection/analysis, Web-based), where the centralizing, unification and pre-diagnosis algorithm running will be performed, as well as the creation of alerts based on results obtained from received investigations;
(iii) In the permanent monitoring center, the specialist doctor investigates the results with possible alerts stemming from highlighted probable pathologies resulting from the pre-processing’s run on the central server, as is shown in Figure 2.

*Workflow description*

1. Creating a new patient form in the application in the FD (family doctor) module if the patient has not been registered beforehand.
2. Connecting the ECG equipment to the patient, controlling the ECG device through the FD module in order to complete the ECG investigation and collect the results in the local data base;
3. Visualizing the results of the investigation in the FD module (ECG graph);
4. Transmitting information regarding the patient (identification data, family doctor) and the results of the investigation to the central server;
5. Receive and store information on the central server;
6. Run automated analysis algorithms on the central server;
7. Showing information + automated analysis results + alerts in the monitoring screen/specialist doctor.

*Automated pre-diagnosis*

The functionality allows the doctor to perform a series of automated tests, that will ultimately result in annotations on the graph, in order to help the specialist doctor to better diagnose.

The application has a pre-diagnosis analysis, the classification of cardiac beats and viewing according to a predetermined classification.

By clicking on a button in the specialist doctor menu, the special signal line will be given annotations in relation to several pre-determined classes:

- Atrial extra systole = 0
- Normal beat = 1
- Left branch block = 2
- Right-left branch block = 3
- Ventricular extra systole = 4
- Aberrant intra-ventricular conduction = 6
- Stimulated ventricular rhythm = 8
- Fusion beat = 9

In a future version the following pre-diagnosis analyses will also be implemented:

- R wave detection.
- Computing the cardiac frequency with alert creation at frequencies larger than 130 beats per minute or lower than 40 beats per minute. An error in cardiac frequency calculus of ±10 beats/minute is admitted.
- Computing the cardiac rhythm variation on a variable number of cardiac complexes. Alerts will be shown on a variation lower or higher than 20% of the cardiac rhythm and the graphical marking of the area.
- Complex QRS detection. For each recording, a maximum of nine complex QRS misses will be acceptable.
- Computing the median of the QRS complex. Alert generation at a median value of the QRS complex duration higher than 120 milliseconds.

This software component has been created as a desktop application that will be run on one or more computers at the monitoring center. Through this application, the specialist doctor has instant access to information regarding the medical form, current ECG investigation, and a complete history of past ECG investigations performed on a patient, regardless of the patient’s location or the previous family doctor. The specialist doctor, through this component, gains access to visualizing and analysis instruments to be applied to the investigation result. Based on these, the specialist doctor may formulate recommendations or alerts, which can be sent instantly to the family doctor’s office

On the investigation page, the family doctor can select the desired measurement instrument depending on time and signal strength. This can be achieved by checking the measurement box. Press the left mouse button and hold to select the desired section to be measured.

## 3. Experimental Model of the Wearable Heart Monitoring Inductive Sensor

The wearable sensor designs require some critical issues to be achieved as: Very low power consumption, small sizes, data storage, and data transfer capabilities, local data processing, and durability. The fulfillment of these conditions gives the portable sensors the quality of “wear and forget” devices.

The classification of the body movement intensity is difficult to be performed during the daily activity. Therefore, the results obtained during “slow motion”, as they are presented in [31], are encouraging but they are not good enough to ensure a proper monitoring for all activities, this is one reason why we recommend to use such sensors for the repose and sleep activities only.

**Figure 3:**
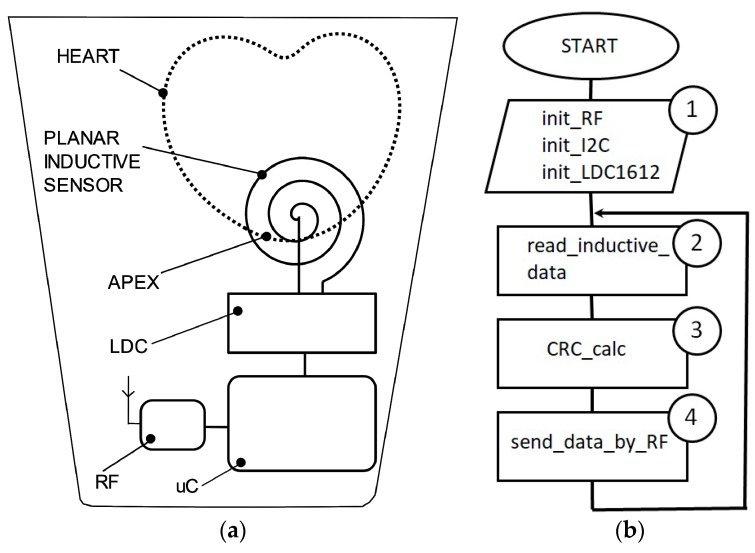
Experimental model block scheme: (**a**) Flowchart of the embedded software of inductive data acquisition and data transfer (**b**).

An LDC1612 circuit is used as inductance-to-digital convertor, as it has a resolution of 28 bits and is able to gather inductance data through two channels. The interface between LDC1612 and a host microcontroller is performed by I2C communication protocol (Figure 3a).

The architecture of the experimental model consists of a microcontroller, an LDC convertor, and a coil implemented in a planar configuration. In this version of implementation, the coil is attached on the fabric in front of the apex. The maximum effect of the heart activity can be detected in this thoracic region.

An L-C resonant is the core of the inductive sensor circuit that induces an EM field and its intensity is determined by inductance, capacitance, and frequency values. An object that is in the proximity of the sensor has an influence on it, according to the electromagnetic properties [29].

We consider a planar spiral to be an optimal shape of the inductance in order to be easily inserted in the fabric of the clothes. A planar spiral inductance is usually found in RF circuits [30] with several profiles: Square, circular, or polygonal. One expression of the planar spiral inductance according to [31] is:

L = K_1 μ_0 (N^2 D_avg)/(1 + K_2 φ)

where:

L = inductance (nH)
D_avg = average diameter of coil (mm)
K_1, K_2 = layout coefficients
φ = fill factor
N = number of turns”

LDC1612 is a high resolution (28 bits) inductance-to-number convertor. The interface between the circuit and the microcontroller unit is performed by the I2C interface. The functionality is based on measuring the oscillation frequency of multiple LC resonators [31]. LDC1612 meets the parametric conditions required by a wearable device. The current consumption in sleep mode is under one microampere and in normal mode it is 1.5 milliampere. The range of power supply is 2.7–3.6 V, which can be powered by a long life or rechargeable lithium-ion battery. The data-rate of I2C interface is 400 kbps and the data-rate conversion is about 4 kSPS [31,32,33,34,35].

ADuCRF101 is a microcontroller unit that has a CORTEX M3 core incorporated and a configurable RF module. The current consumption in sleep mode is under one microampere.

**Figure 4:**
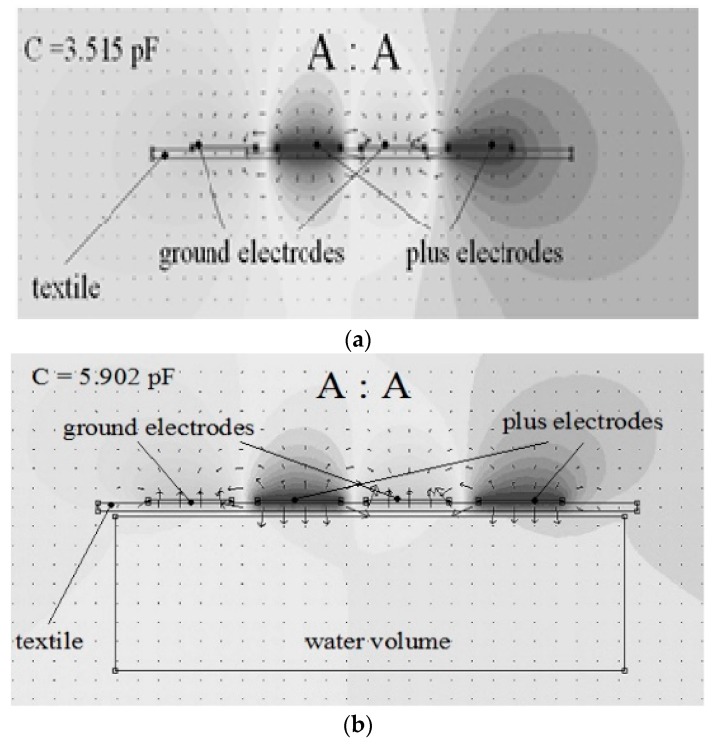
The electric field intensity of the sensor electrodes, simulated with Beladraw 1.0 software for an interdigital capacitance without water probe [24] (**a**) The electric field intensity of the sensor electrodes, simulated with Beladraw 1.0 software for an interdigital capacitance with water probe [24] (**b**).

The source code of the microcontroller is described in C language and is compiled by Keil tool. The program execution follows the successive operations described in the flowchart (Figure 4b), respectively. Label 1 represents the functions of initialization for the ADuCRF101 microcontroller and for the LDC1612 convertor. The setting of the microcontroller consists of: Clock frequency to 4 MHz, RF frequency: 868 MHz, RF data-rate: 300 kbps, length of the data stream: 24 bytes. LDC1612 is set up to work in continuous mode. Inductive data are stored in the internal RAM location as vectors having a length of 24 bytes.

The data are sent to a data concentrator that is connected to a laptop and are saved as text format. In order to avoid errors during the data transfer, a CRC algorithm is used. All the circuits are powered by a 3.6 V battery of and an energy capacitance of 2700 mAh. The average current during data acquisition is about 6 mA and during data transfer, it is about 36 mA (for an RF power of 13 dBm).

The human biological tissues are mostly composed of water. The heart and the brain consist of about 73% water, the lungs 83% and the bones contain about 30% water [34]. Based on the influence of water on the electrical and electromagnetically field parameters, proximity sensors were conceived. The parameter of the electrical field that is strongly influenced by water is the electrical permittivity, respectively εr. The presence of a water body in the proximity of capacitance will strongly influence it, increasing its value.

Reference [24] presents the influence of a water probe on a planar capacitive sensor as simulation results using Beladraw 1.0 software (Figure 4a,b).

When a water probe is near electrodes, at a distance of 1 mm, the capacitance value increases to 5.902 pF. The proximity capacitive sensors have a very good sensitivity, but a disadvantage is the influence of environmental humidity on the measurement accuracy. In clothes applications, for example, perspiration is a factor to be considered.

**Figure 5:**
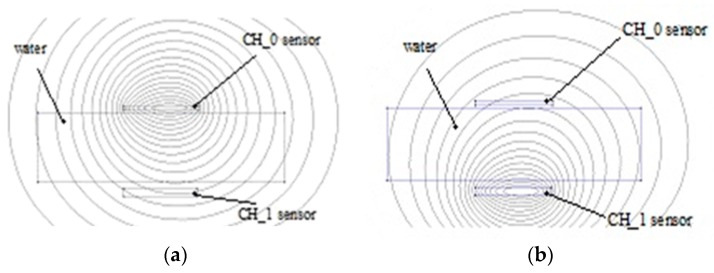
Inductive proximity sensor: Simulated model. CH_0-activated (**a**) Inductive proximity sensor: simulated model, CH_1-activated (**b**).

In the case of the electromagnetic field, the influences of various materials that are found are increasing, decreasing or have no influence on the permeability parameter µr. The water diamagnetic property will have as an effect a decreasing of the inductance value, therefore, when the sensor approaches the tissues the value is less than when the sensor is placed at a larger distance (Figure 5a).

Activating CH_0 determines the value of the inductance by the femm simulator as 3.45406 µH and when CH_1 sensor is activated (Figure 5a,b) the value of its inductance is 3.50515 µH. The water has a negative permeability due to its diamagnetic properties.

## 4. Mobile ECG Device

The main component of the portable device is the specialized integrated circuit ADAS1000 made by Analog Devices, which can work with 3, 4 or 10 electrodes (in a master–slave configuration) in order to obtain results on 3, 5 or 12 leads. The block diagram includes a microcontroller, the ADAS1000 circuit, a GSM/GPRS communication module, an accelerometer, and a temperature sensor.

The acceleration sensor detects if the patient is moving in order to make a correlation between the ECG data and the movement. The temperature sensor shows whether the patient has a fever. Beside the ECG information, the ADAS1000-integrated circuit also generates data on breathing, which is useful for the detection of sleep apnea.

During the ECG data reading, data communication is off, so that the signal input is not affected. After the reading interval of 10 s, the communication module GSM SIM900 is turned on and initialized. It contains a SIM card from a GSM data service provider. The microcontroller AduCRF101 from the portable device communicates with the GSM SIM 900 modem by means of a serial port and of GSM AT commands.

The SIM900 modem is a 2G GPRS mobile data module functioning in the GSM850 MHz, EGSM900 MHz, DCS1800 and PCS1900 frequency bands. The data read by the ADAS1000 circuit is processed by the microcontroller in order to be stored temporarily (RAM) or permanently (FLASH), and then they are sent regularly through the GSM/GPRS data communication in the cloud to a GreenCardio© server by means of the FTP data protocol. If there is no Internet connection, the data is saved in internal FLASH memory and it is sent to the central server when the Internet connection is available.

The data is stored in a *.xml file. Its name indicates the day and the time of the data input, for example: 2018_07_26-14_01_20.xml. Within the GreenCardio© server, the data is processed individually for each patient and analyzed by a physician.

Such a file is presented below:

<?xmlversion=“1.0” encoding=“ISO-8859-1”?>

<!DOCTYPE RestingECG SYSTEM “rest.dtd”>

<RestingECG>

<PatientID>Maria Popescu</PatientID>

<DateofBirth>01-08-1982</DateofBirth>

<Gender>Female</Gender>

<Waveform>

<WaveformType>Rhythm</WaveformType>

<NumberofLeads>1</NumberofLeads>

<SampleType>CONTINUOUS_SAMPLES</SampleType>

<SampleBase>500</SampleBase>

<Battery>Stare incarcare=0, Nivelbaterie=90%, Tensiunebaterie=4,061V</Battery>

<LeadData>

<LeadAmplitudeUnitsPerBit>2.84</LeadAmplitudeUnitsPerBit>

<LeadID>1</LeadID>

<LeadDataCRC32>4291035169</LeadDataCRC32>

<WaveFormData>FF 0000 0000 0000 FF 0000 0000 0000 FF 0000 0000 0000 FF 7FAD 8053 7FB6 FF 7F79 804D 7F6C FF 7E33 802A 7C0D 802D 7C0E FF 7D02 802D 7C0E FF 7D02 802D 7C0F FF 7D02 802D 7C0F FF 7D02 802D 7C0F00000</WaveFormData>

</LeadData>

</Waveform>

</RestingECG>

**Figure 6:**
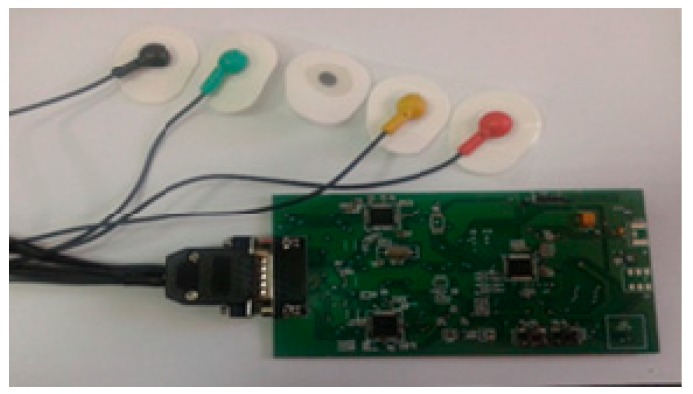
Experimental model using an ADAS1000 demo-board.

The above figure presents an ECG signal reading device made with two ADAS1000-integrated circuits connected as master and slave, which allows the representation of 12 derivations (member derivations and precordial derivations), as observed in Figure 6.

## 5. Data Acquisition

**Figure 7:**
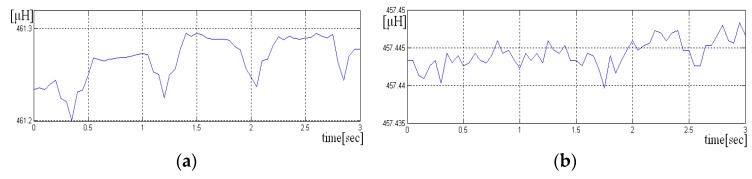
Sequence of three seconds from an acquisition with sensor pressed. The periodicity of signal is visible. (**a**) Sequence of three seconds from acquisition with sensor not pressed. The periodicity of signal is difficult to be detected (**b**).

Inductive data generated by LDC1612 have 28 bytes size for each sample, but we use only the most significant 16 bits. The average length of each recording was approximately 30 s. The signals were acquired at the sampling frequency of $f_{s}$ = 20 Hz. Two scenarios were used to acquire the signals, one in which the sensor is held “closer” to the chest and the other in which the sensor is not pressed. In the following figure, we have a portion of an acquired signal holding the sensor closer to the chest box, where one can clearly see the periodicity of the heart-generated signal (Figure 7a).

If the sensor is not pressed, the signal has more noise, and the periodicity due to heart activity is less visible. A short sequence of three seconds from such signal is shown in Figure 7a,b. Even if the signal is noisy, it is still possible to detect the heart rate, if the size of the analysis window has more than 10 s.

Acquisitions were also made, in which the subject at first started breathing normally (with an air exhaled at about 3–5 s), and finally holding breath. This, way it was observed that in the first half of the signal, in which the subject does not breathe, the periodicity of the signal associated with the activity of the heart is more visible.

**Figure 8:**
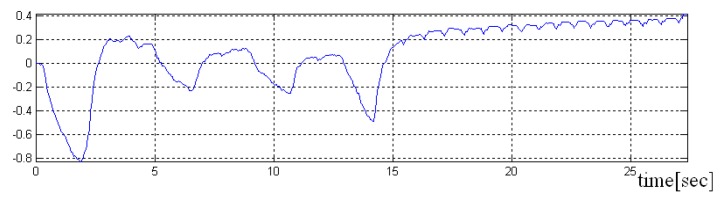
Acquisition with normal breath in the first part, and without breath in the second part of the signal.

**Figure 9:**
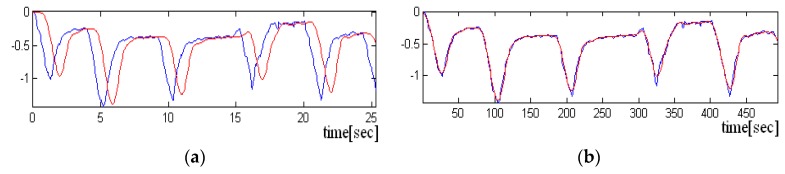
Representation of the delay between the input signal (with blue) and the filtered signal (with red) (**a**) Representation of the overlap of the input signal and the filtered signal (the delay was eliminated) (**b**).

**Figure 10:**
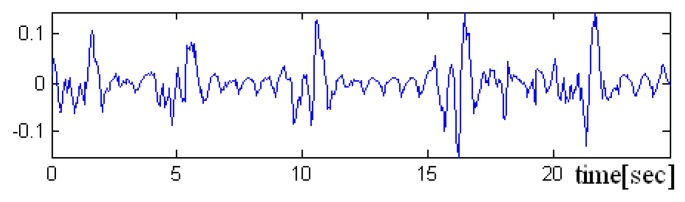
Representation of the difference signal (which mainly contains the periodic heart signal).

In the Figure 8, Figure 9 and Figure 10 a pre-processed signal, filtering and normalization operators that were applied on this signal are presented, therefore, the values of interval was changed and the signal amplitude is not expressed in μH as it is adimensional. In future research activity, we intend to use DWT (discrete wavelet transform), by changing the detailed coefficients that contain also the noise, respectively, without altering the approximate coefficient as in work [36]. A predictive neural network could be implemented in order to increase the robustness to noise.

Because the heart rate detected for both parts of the signal was approximately the same, it was concluded that the implemented algorithm is as effective for both situations.

## 6. Data Processing and Results

Data processing consists of the implementation of an algorithm in order to extract two components, the respiration components, and the heart rate component from the same signal generated by the LDC circuit. Because the inductive sensor incorporates both these signals, $s=s_{B}+s_{H}$, we should find a filtering method in order to correctly split the target signals.

The algorithm is:

**Step 1**: Removing the amplitude offset from signal *s*

$$s_{n}=s_{n}-mean(s)$$

**Step 2:** Applying a low pass filter LPF with a cut-off frequency of 1 Hz, in order to extract the respiration components $s_{B}$:

$$s_{n}^{*}=\sum_{k=0}^{M-1}s_{n-k}\cdot b_{k}+\sum_{j=1}^{M-1}s_{n-j}^{*}\cdot a_{j},\;s_{n}^{*}≅s_{B}$$

The coefficients of the digital filter $a_{j}$, $b_{k}$ are generated by Matlab and according to a filter that belongs to the Butterworth family having *M* = 7 order and the cut-off frequency as mentioned above, at 1 Hz.

The complete equation of the ARMA filter (autoregressive moving average) includes in the formula a recurrent part AR, that induces a delay in the filtered signal $s^{*}$.

**Step 3:** the delay between the inductive signal s and filtered signal $s^{*}$ is determined using the AMDF (average magnitude difference function) method and the correlation function.

- initializing with zero of the vectors $C_{XY}$ and $D_{XY}$

For *k* = 0: length_s-1

$$C_{XY}[k]=\sum_{n=0}^{N-k}s[n]\cdot s^{*}[n+k],\;k=0,\ldots ,N-1$$

$$D_{XY}[k]=\sum_{n=0}^{N-k}s[n]-s^{*}[n+k]$$

We denominated the number of samples/the length of the inductive signal (input signal) with *N*.

The $C_{XY}$ and $D_{XY}$ vectors calculation could be limited only for a number of values done by maximum delay where the signal could arrive and not on all length of *s* signal.

The offset is determined based on the global maximum position of the correlation function $C_{XY}$, respectively on the minimum global position from $D_{XY}$ vector of AMDF.

$$offset(1)=\underset{k}{\min}D_{XY}$$

$$offset(2)=\underset{k}{\max}C_{XY}$$

Depending on the input signal, any of the two methods can determine better the offset. the time lag which has led to the best overlap is chosen (with a minimum difference).

$$\mathrm{if}\;mean(d_{1})<mean(d_{2}),$$

$$offset=offset(1),\;d=d_{1}$$

$$\mathrm{else}\;offset=offset(2),\;d=d_{2}$$

The differences in signals between input signal *s* and the filtered signal $s^{*}$ were calculated taking into account both delays.

$$d_{j}=\sum_{n=0}^{N-offset1}\left(s[n]-s^{*}[n-offset(j)\right],\;j=\{1,\;2\}$$

Removing the respiration components $s_{B}$ from the input signal will keep the heart rate component $s_{H}≅d$

**Step 4.** The period T of the difference signal is estimated, using the autocorrelation function. The signal period T is considered to estimate the heart rate.

For *k* = 0: length_d-1

$$C_{XX}[k]=\sum_{n=0}^{N-k}d[n]\cdot d[n+k]$$

Only local peaks between $T_{\min}$ and $T_{\max}$ are taken into account. These limits are set by the minimum heart rate considered to be 40 bpm and the maximum value set at 200 bpm:

$$T_{\min}=60/200\cdot f_{s},\;T_{\max}=60/40\cdot f_{s}$$

where $f_{s}=20\;\mathrm{Hz}$ is the signal sampling frequency.

The signal period is given by the local maximum position between the two previously set limits.

$$T=index\_of\underset{k}{\max}C_{XX}[k]k\in \left[T_{\min},T_{\max}\right]$$

**Step 5.** The cardiac rhythm is estimated in the current analysis window as being:

$$bpm=60/T\cdot f_{s}$$

Because the filtered signal $s^{*}$ approximates the signal due to breathing very well, in the signal difference $d=s-s^{*}≅(s_{H}+s_{B})-s_{B}$ will be found the signal generated by the activity of the heart $s_{H}$, but also some high-frequency components of the signal $s_{B}$.

In addition to these high-frequency components, a noise is added that resembles uniform noise for recordings in which the sensor is not pressed/held close to the chest, but with all these impediments, heart rate detection can be performed. For these recordings, as noted above, the duration of the analysis window should be longer than 10 s.

The algorithm proposed by us has the advantage of simultaneously extracting both components, both of which are of interest because they can provide additional information about people suffering from certain diseases (e.g., apnea). The QRS complexes detection process is difficult, not only because of the physiological variability of QRS complexes, but also because of the different types of noise that an ECG signal contains.

The noise sources include muscle artifacts due to the motion of the electrodes, interference from power lines, the change in the baseline, T-waves with a high frequency similar to the QRS complexes [37,38,39,40]. The artifacts are considered as external noises induced by muscle activities. The term “muscle noise artifact” is also found in other papers, an example could be [39].

Once the signal has been filtered, we applied a set of rules to correct QRS complexes detection [37,38]. In order to compare the heart rate values obtained with our system, the ECG signal was acquired in parallel using a BIOPAC system. The hardware modules used are the MP150A-CE data acquisition unit and the ECG100C, electrocardiogram amplifier modules with reusable EL258 electrodes. The MP150A allows simultaneous 16-channel acquisition with a sampling frequency up to a maximum of 200 kHz.

The sampling frequency we used was *f_s_* = 200 Hz and the signals had a length of 30 s. The ECG100C module is a single channel and allows a gain between 500 and 5000. It is equipped with a high pass filter over frequencies of 0.05 Hz or 1 Hz, respectively, with a 35 Hz or 150 Hz low pass filter. It also has a notch filter to eliminate the 50 Hz frequency of the electrical network. Electrodes of the ECG100C mode were connected to measure Lead I (the two active electrodes being placed on the right arm and the stand arm, and the table electrode at the right foot). The acquisition was performed with 1Hz HPF in order to stabilize the baseline ECG. At this frequency, P and T waves may have slightly lower amplitude, but the QRS complex is not affected. Since the purpose of acquisition of these signals was to detect QRS peaks and to verify that the heart rate values obtained by the inductive sensor system we preferred this cutting frequency for HPF instead of 0.05 Hz. For the LPF filter the frequency 35 Hz ensures better noise elimination.

Even if these hardware filters were used to make the acquisition, additional software filters were required because some of the acquired signals had ECG baseline fluctuations (and the algorithm needed to be a straight line), respectively, the noise evenly high enough, the level of this noise also depending on how good the contact between the electrodes and the skin is.

The implemented algorithm has three main stages:

- baseline determination,
- detection of QRS complex,
- removing other information (where P and T).

## 7. Conclusions

The method of using inductive sensors is useful to monitor the data rate and the respiration, as vital signs, incorporating the “wear and forget” device in the clothes. The data rate signal and the respiration signal are extracted from the same output signal of inductance-to-number convertor.

The method and the wearable sensors incorporated in the clothes are suitable to monitor physiological moving parameters that have small amplitudes (e.g., mechanical heart activity, respiration, tremor, pulse, etc.) only if the body is in a repose position. Any muscle activity will induce artifacts in the signal generated by the sensors. Their influence will be proportional to the intensity of body movement. We take into account using an acceleration sensor in order to validate the inductive signal (when the body is in repose, without muscle activity) and to eliminate the artefacts based on correlation with the acceleration signal.

The body motion during data sampling has a large influence on the inductive signal inducing its motion artifacts and we concluded that this method is not suitable to be used in daily activity. During the daily activity it is difficult to perform a classification of the body movement intensity. Therefore, the results obtained during “slow motion” are encouraging but they are not good enough to ensure proper monitoring for all activities. This is one reason why we recommend using such sensors for the repose and sleep activities only.

The theme of our paper is to extract from the same signal, generated by the inductive sensor, two parameters: Respiration and heart rate. For the statistical comparison, we intend to use the representing data using Bland–Altman plots. In the future work, we propose to correlate the inductive sensors data with ECG data and acceleration data in order to implement other models for intelligent textiles, and to improve the GreenCardio System©.
